# An Interesting Case Involving the Reputation of a Medical Brother

**Published:** 1885-10-20

**Authors:** P. S. Verdery

**Affiliations:** Douglassville, Ga.


### Southern Medical Record.

EDITORS:

THOMAS S. POWELL, M.D. R. C. WORD, M.D.

*R. C. WORD, M.D., Managing Editor.*

☞All Communications and Letters on Business connected with the RECORD must be addressed to the Managing Editor.

VOL. XV. ATLANTA, GA., OCTOBER 20, 1885. NO. 10.

### ORIGINAL AND SELECTED ARTICLES.

### AN INTERESTING CASE INVOLVING THE REPUTATION OF A MEDICAL BROTHER.

BY P. S. VERDERY, M. D., OF DOUGLASSVILLE, GA.

*Editors of the Southern Medical Record:*

I have just had a singular and painful experience in a case to which I wish to call your attention. Knowing, as we all do, the high standing of your journal in the Southern States, any opinion from you will be most conclusive:

THE CASE.

Was called on Sunday, August 2, to Willie Rives, aged sixteen, of Douglass County, Ga., and found that he had been sick since the Thursday before. The attending physician—Dr. Rhodes, of Chapel Hill—with whom I was called to consult, and help treat the case, being in doubt, I pronounced it a case of Typhoid Entiritis in a most aggravated form. His tongue was heavily coated down the centre, very sharp pointed, and red to scarlet on the edges; his respiration was most labored, and alarmingly slow; his nervous symptoms were those of prostration, his tongue being protruded with difficulty, and very tremulous, and his countenance wore a most anxious and painful look; his abdominal symptoms were serious, there being a good deal of tympanitis, and the tenderness was so great that he could not tolerate percussion ; in fact, a slight tap with the fingers on the distant walls of the thorax caused acute pain all through the abdomen, particularly in the region of the illio-cœcal valve ; pulse, 95 to 100, and full. His body, on the breast and back, also his face, was covered with a reddish-purplish flush, not the petechia of fever exactly, but resembling it very much, with here and there a small vesicle, and in four or five vesicles the serum had turned to pus.

On the day that he was taken sick he had eaten heartily of water-melons, fruits, etc., and his attack commenced in the night with what appeared to be an attack of indigestion (to Dr. Rhodes, who, on arriving, very properly proceeded to empty the patient's stomach and bowels with oil, enemas, etc.). He was then put by Dr. Rhodes on quinine, gr. iv, with about one ounce of whisky, every two hours, with morphine sufficient to relieve pain and restlessness. After consulting, we put him on the following : Iodid. potass, gr. ii ; carbolic acid, gtt. ii, every four hours, with bromid potass, gr. xv ; sweet spts nitre, f℥, every four hours, alternate ; and, as his stomach seemed to revolt at morphine, it was administered hypodermically when needed to relieve pain. The above prescriptions were not to be given at the hours set if the patient was asleep and resting well. As the tenderness over the illeo-cœcal valve appeared so severe, I, contrary to my rule in that disease, ordered a small blister over that spot. The quinine was stopped, and the whisky used only as we thought was indicated.

On the next two days his treatment was not materially changed, nor was his condition. On Wednesday I failed to see the patient. but on the next day, when I returned, I found him decidedly worse, His tongue was brown and dry ; teeth with sordes ; pulse, 120, and his abdomen distended so tightly that the swelling had extended far above the ensiform cartilage, and so tender that any thorough examination was impossible, but the pain was refered mainly to the region of the valve. The breathing had become so slow and labored that, at times, it seemed as if it was insufficient to sustain life ; his bowels had become constipated. Enemas were then tried repeatedly, and then a large dose of castor oil ; that failing to produce any movement, I then commenced to give podophyllin and rheubarb, so that, in all, he took about 3 ounces of syr. Rhei aro, and about 4 grains of podophyllin. They failed to act, and noticing that the gastric distress grew worse, and that the stomach proper was distended as well as the abdomen, I commenced to give bi-carb. soda in teaspoonful doses. This caused the patient to vomit and evict gas from his stomach, which appeared to reduce the distension in the epigastrium, but gave no relief to his abdominal symptoms. The matter vomited was of a nature that, in a practice of seventeen years, I had never seen anything like it. It was the consistency and color of bile, but the odor was so hideously offensive that those around the bed could hardly remain at their posts, and like to have fainted. I can compare the smell to nothing more like it than the effluvia from a human body in the worst stages of decay. All the friends thought, sure "he was rotten inside, and bound to die." I continued the soda until the contents of the stomach changed their smell; the patient, in the meantime, was bathed in a cold and profuse perspiration, his pulse increasing in frequency and decreasing in volume, while whisky and morphine were used as indicated or could be tolerated.

That condition of affairs went on through Thursday, Friday and Friday night. His pulse had now reached 145, and his breathing was no better, and it was evident to all that, unless relief came speedily, death would ensue. Under these circumstances, and as it was *certain* that he could not live in his then condition, and he might stand a little chance if the distension in his bowels was relieved, I gave him the following dose of Croton oil: I made 4 drops into four pills, and gave him two of them, and then directed (as I had to leave) that, if his bowels did not move in six hours, to give the other two pills. When the time arrived the other two pills were given, much against the judgment (?) of the surrounding friends. I returned and found the same state of affairs as when I left. . . .

The second dose of oil had been given. Night had closed in, and the weary parents and watchers retired, expecting to be called to see him die before the dawn. At 2 o'clock a. m. there came a change. Eleven hours from the last dose of Croton oil his bowels gently moved. At that time his heart was feebly clicking 145 to the minute; cold perspiration bathed his face, arms and hands, and his labored breathing was as painful to witness as it had been. After the action of the oil, I counted his pulse; it had gone down a little. In an hour his bowels moved again; the perspiration then ceased. At daylight they moved again; his pulse then indicated 100; the perspiration was all gone; warmth had returned to his surface; his countenance assumed a more natural look, and it was evident to all that the crisis was passed!

About the time he took the Croton oil he commenced to break out with an eruption which, to me, looked like the vesicles he had on his body, scattered over his breast and back, where the reddishpurple eruption was; his face also broke out with the same pustulous eruption. It would come in the same way that a vesicle usually forms, then the serum would turn to pus in about twelve hours from the first appearance of the trouble. I decided that the eruption was caused by the vitiated condition of his blood, and that the disease, while it resembled typhoid fever, resembled just as much typhus, as all his other symptoms were too rapid for pure typhoid. I also decided that the disease was contagious in the same sense that typhoid or typhus fever is, and so stated, and advised and commended the use of disinfectants. I thought that the matter vomited was of such an extraordinary character that it had been partly absorbed and poisoned his blood; and, hence, the eruption. Certain there must have been extra poisons, or extra amount of fever poison generated, that produced such discoloration of the skin, which finally ended in eruptions; and on this point I would like your opinion. Had I the right to mine? Just as the case reached this stage I was summoned home to watch at the bedside of my own son, eight years old, who died of albuminuria, and was obliged to engage another physician to take the case until I could return. After my child was dead and buried, I returned to resume my charge (being gone about thirty-six hours), and found that the family and the people generally had lost all confidence in me, and the patient's father made me the proposition that I continue to attend the case in connection with the physician who had attended it for me during my temporary absence at *half price, the other doctor working for the same figure!* I was astounded, and refused to have anything further to do with the case, the other physician taking it and treating it until death claimed the patient, which was in about a week or ten days from his resuming charge.

On inquiry, I find the main cause of complaint is that the *four drops* of Croton oil was the cause of his breaking out all over his back, breast and face; that it had done him a great deal of harm, and ultimately caused his death. I have consulted all the physicians out here, and some in Atlanta, and all, without a single exception, pronounce the idea absurd. I have consulted all the leading written authorities in my reach, and cannot find a single word to sustain any such idea. I have, on several occasions in my life, seen from four to ten times that amount given, and never saw any such result, whether it acted or not. As well might a man say an ordinary dose of cantharides or tartar emetic, or any other drug that irritates, vesicates or pustulates the skin would cause such an eruption; and in this case you would have to ignore the fact that fevers frequently are associated with eruptions of various kinds.

It is the belief among the people generally that there is something terrible about Croton oil, and that in any size dose it is a most virulent poison ; in any size dose its administration is fraught with imminent danger to the patient. Indeed, out here in my county, were you to propose to give a dose of Croton oil to a man he would at once decide you wanted to kill him ; and one old man went so far as to tell me that, under no circumstances would he allow me to give a single drop to him or any of his family.

Advantage has been taken on account of the popular prejudice and ignorance about Croton oil, to do me a most decided injury, and to ruin my reputation as a physician. Indeed, unless I can establish by you, and such leading physicians as Atlanta contains, that my treatment of that case was right, and that there was no authority whatever for saying Croton oil did in that case what it has never been known to do in the two hundred years it has been used in civilized practice, I cannot hope to regain the position as a physician that I occupied in this county, and that I have labored hard and HONORABLY for ten years to attain. I don't want to have to submit to the verdict, that "Nobody but a fool or a drunk man would have given a dose of Croton oil." *I* know I am right, and now, sirs, I appeal to you, through the pages of THE RECORD, to give weight to my assertion. My name is familiar in Georgia, and on my *merits alone* as a physician would I wish to rise or prosper, and I had rather, to-morrow, work at fifty cents a day as a laborer, than to gain popularity on a *single case* by false representations.

As to my standing as a physician, I refer you to all my brother physicians in this and adjoining counties.

[WE are asked by the writer of the above for our written opinion as to the justice of the charge referred to in the case stated—to wit, that his use of the Croton oil was the cause of the patient's death. He does not say that the imputation was made by the physician who succeeded to the treatment of the case. We would hope, for the honor of the profession, that such was not the fact. Feeling it a duty, which every medical man owes to his brethren in the profession when their professional reputation is unjustly assailed, we cheerfully gave him the written opinion as requested, of which the following is a copy :

Touching the question presented by Dr. P. S. Verdery, as to whether the internal administration of two drops of Croton oil once repeated will cause an eruption upon the external skin, we hesitate not to state that we have never known, read or heard of any such result to follow its use as an internal remedy ; and as to the case described by Dr. Verdery, the complaint made that the Croton oil caused the death of the patient, is, in our opinion, utterly devoid of any good reason to sustain it.—EDITORS OF THE RECORD.]

